# Engineering rank queries on bit vectors and strings

**DOI:** 10.1186/s13015-025-00291-9

**Published:** 2025-12-11

**Authors:** Simon Gene Gottlieb, Knut Reinert

**Affiliations:** 1 Informatik & Mathematik, Freie Universität Berlin, Takustraße 9, 14195 Berlin, Germany; 2 Efficient Algorithms for Omics Data Group, Max-Planck-Institute for Molecular Genetics, 14195 Berlin, Germany

**Keywords:** Bit vector, Rank support, String, FM-Index, SIMD

## Abstract

Adding rank support to strings over a fixed-sized alphabet has numerous applications. Prominent among those is the (bidirectional) FM-Index which is commonly utilized to index and analyze genomic data. At its core lies the *rank* operation on the Burrows-Wheeler-Transform (BWT) which, given a position in the BWT and a character, answers how often the specified character appears from the start to that position. Implementing those rank queries is usually based on bit vectors with rank support. In this work, we discuss three implementation improvements. First, a novel approach named *paired-blocks* that reduces the space overhead of the support structure by half to a total of only $1.6\%$. Second, a method for masking bits for the population count (also known as popcount) which greatly improves the runtime of 512-bit wide blocks in conjunction with AVX512 SIMD extensions. Third, a revised method for EPR-dictionaries (Pockrandt et al. in International conference on research in computational molecular biology. Springer, New York, 2017) called *flattened bit vectors* (fBV) with less space consumption and faster rank operations on strings, which is competitive in size and depending on the parameters between $2\times$ and $9\times$ faster than Wavelet Trees (Gog et al. in 13th International Symposium on Experimental Algorithms. Springer, New York, 2014).

## Introduction

In genome data analysis, large amounts of genetic sequences are processed. Memory usage and execution time are both important considerations. A common application is constructing an FM-Index [2, 3] over a reference genome. The index can be used to locate or count the occurrences of a query sequence and is hence often used for solving the approximate pattern matching problem. Many applications in bioinformatics rely on the FM-Index to compute read alignments [4–17].

The FM-Index can show its advantages especially when handling strings over a small alphabet such as the DNA or amino acid alphabet. A crucial part for the performance of FM-Index implementations lies in the implementation of *rank* operations on strings, which is an internal operation of the FM-Index and a key contributor to space requirements and execution time.

Given a string $S=s_1\ldots s_n$ of length $n=|S|$ and $s_i \in \Sigma$ where $\Sigma$ is a finite set of characters. The *rank* operation takes two arguments. A position *i* and a character *c* and returns the number of characters *c* in the first *i* positions of a string.

$$\begin{aligned} rank(i, c) = |\{j \in [1, i]: s_j = c\}| \end{aligned}$$

A bit vector with rank support addresses the special case of $\Sigma =\{0, 1\}$. Here, the *rank* operation counts the number of 1 s in the first *i* positions (note that the number of 0 s can be inferred trivially).

$$\begin{aligned} rank(i) = \sum _{j=1}^i s_j \end{aligned}$$

The binary case is well-researched. Jacobson [18] showed that by utilizing some auxiliary data structures, bit vector queries can be answered in constant time, with sublinear memory consumption for the auxiliary data structure, even without special hardware support like popcount. The auxiliary data structures are usually two arrays named *blocks* and *superblocks* which contain prefix sums.

The bits are grouped into superblocks of $w_1$ bits. All superblocks are considered a single layer of auxiliary data. Each superblock is annotated with the number of 1 bits that have appeared in all superblocks prior to this one. Additionally, the bits are also grouped into blocks of $w_2$ bits, which form an additional layer. Each block is annotated with the number of 1 bits that have appeared in the blocks prior to this one until the beginning of the last superblocks. Superblocks cover several blocks and require that $w_1$ is a multiple of $w_2$. It is possible to implement only a single layer (removing the blocks) or more than two layers. To achieve sublinear memory consumption two or more layers of auxiliary data must be used and carefully selected $w_1$ and $w_2$ values like $w_2 = w_1^2$ and $w_1 =\frac{1}{2}\log _2 n$ for a string of length *n* must be chosen.

In practice, the block sizes are chosen such that they meet practical considerations about word boundaries and cache sizes as well as the tradeoff between the space overhead of the auxiliary data structures (in relation to the *n* bit of the bit vector) and the time to answer rank queries.

In 2008 Vigna [19] suggested a compact data structure that reduces cache access to a single access by interleaving the auxiliary structures of the layers. This implementation is referred to as *rank9* reflecting the 9-bit width of the prefix sum values in the first layer. It requires a space overhead of 25%.

In 2013 Zhou et al. [20] reduced the 25% overhead to an impressive 3.125% space overhead. This is achieved by using 512-bit wide blocks on the lowest layer.

In 2022 Kurpicz [21] expanded on Zhou et al. ideas and improved the run time properties of the *rank* operation by interleaving the values and making the structure cache-friendly while maintaining the 3.125% overhead.

At the same time, further research was made into compressed bit vectors [22–24] which achieve improved space usage dependent on the content of the bit vector. The approach by Grabowski and Raniszewski [23] as well as the approach by Pibiri and Kanda [24] have direct comparison against the *rank9* implementation by Vigna [19]. In both studies *rank9* has better run times. These implementation have to be tested with varying compressibility for proper evaluation. Because of this special type of setting, we did not include these studies in our research.

The FM-Index, introduced by Ferragina & Manzini, requires a *rank* operation on strings over larger alphabets. One way to achieve the required *rank* operation on strings is to use a bit vector for each character of the alphabet. Each bit vector has the same length as the strings and indicates with a 1 if that specific character is available. The space complexity of this solution (in the following called *multiple bit vector (mBV))* is linearly dependent on the length of the bit vector and the size of the alphabet, namely $O(n \cdot |\Sigma |)$, while the time for a *rank* query is *O*(1). Hence this solution is only viable for very small alphabets.

In 2003, Grossi et al. [25] introduced the concept of Wavelet Trees which was further refined by Ferragina et al. [26] to answer *rank* operation while only requiring $O(n \cdot \log |\Sigma |)$ space. This is done by creating a binary tree with each character of the alphabet as a leaf. Each node of the tree splits the alphabet in half, which reports on how many characters are in the upper or lower half. However, each *rank* operation requires $O(\log |\Sigma |)$ steps since it traverses the tree from the root to a leaf. The nature of the Wavelet Trees and the local independence of each access on each node, have a high impact on the run time. This implementation (in the following called *wavelet*) trades space for speed.

The mBV and wavelet implementations can be seen as two extreme points in the quest for achieving a good combination of space overhead and run time. mBV is very fast, but uses a lot of memory while wavelet is slow, but space-efficient. Research for practical implementations has been ongoing.

In 2016, Pockrandt et al. [1] suggested an implementation with a query time of *O*(1). It requires more memory than wavelet, but less than a bit vector based implementation. The memory requirement increases to $O(n\cdot \log |\Sigma |) + o(n\cdot |\Sigma | \cdot \log |\Sigma |)$ space.

Anderson & Wheeler [27] describe a memory layout specifically designed to improve the execution time of an FM-Index when using biological alphabets like DNA or amino acids. By scattering the bits of a single symbol across multiple machine words, they can utilize the AVX2 instruction set, which can perform bitwise operations over 256 bits at once. Their implementation has similarities to a multi bit vector implementation which uses a single layer and flattens the bits inside a block into a compact form, requiring $O(n\cdot \log |\Sigma |) + o(n\cdot |\Sigma |)$ bits.

In our research we want to suggest three improvements: Firstly, we suggest a novel improvement to the usage of bit vectors for rank support that halves the space overhead requirements. We refer to this layout as *paired-blocks*. Secondly, we discuss improvements to performing population counts for 512-bit wide blocks. Thirdly, we further improve the memory layout for strings with rank support. We optimize it for space and speed such that it is competitive to all other existing solutions and works for arbitrary alphabet sizes. We call this solution *flattened bit vectors* (fBV).

## Methods

### Paired-blocks

To understand the main part of our *paired-blocks* bit vector idea, we will reiterate how a bit vector with rank support can be implemented with a single layer and demonstrate our improvement. Afterward, we will discuss how this can be transferred to two layers.

#### Single-layer

To enable fast rank operations on bit vectors the bits are grouped into blocks of *w* bits. For each block, we store the prefix sum value of all the 1 bits that are present in the previous blocks in an array *L*0. This reduces a rank operation to only counting the 1 bits in a single block until the requested position and adding this to the value associated with this block. Let *L*0[*i*] denote the *i*-th entry of the prefix sum values. Let *b*[*i*] denote the *i*-th group of bits. Let *b* denote a string of bits then $b_{:i}$ refers to a prefix of length *i* covering the first *i* bits and $b_{i:}$ the suffix starting at the *i*-th bit. Notice that the bit string *b* is equal to concatenating the bit strings $b_{:i}$ and $b_{i+1:}$.

The popcount function *pc*(*b*) counts the 1 bits of the value *b* (i.e. its binary representation).

The values *L*0 are defined as:

1 $$\begin{aligned} L0[i] = \sum _{j=1}^{i-1} pc(b[j]) \end{aligned}$$

To compute *rank* a simple access to *L*0 and *b* has to be made:

2 $$\begin{aligned} i_{l0}&= \left\lfloor \frac{i}{w} \right\rfloor \end{aligned}$$

3 $$\begin{aligned} bits&= b[i_{l0}]_{:i-i_{l0} \cdot w}\end{aligned}$$

4 $$\begin{aligned} rank(i)&= L0[i_{l0}] + pc(bits) \end{aligned}$$

**Fig. 1:**
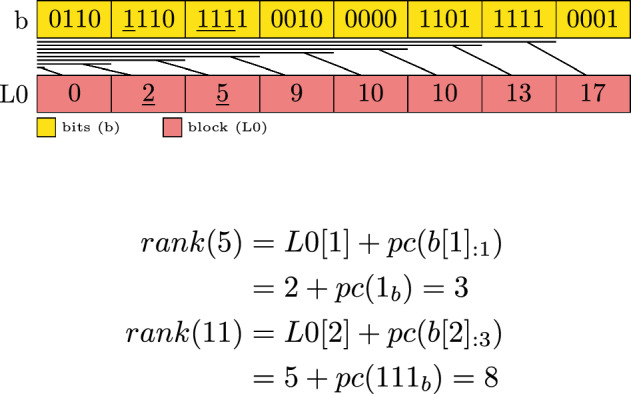
Example of a single layer bit vector with block size $w=4$. The lines between the layers indicate which section of the bits the prefix sums correspond to. Underlined values are accessed in the exemplary rank computation

Typical values for *w* are 64 or 512 for common hardware architectures. Modern CPUs provide a *popcount* instruction that accelerates the count of bits inside a block. To count only the leading bits in a block, it is shifted to the right, which removes the unwanted bits and then a *popcount* is performed resulting in the desired count of 1 bits. Figure 1 demonstrates the rank query for positions 5 and 11. Note that we depict in the figures the bits from left to right for ease of exposition.

Our proposed idea is to *pair* blocks into a *left* and *right* block (for brevity sometimes called *rb*) and store only the associated prefix sum value for a right block. This halves the number of stored values. To retrieve the result for a rank query, we distinguish two cases depending on whether the requested bit position is inside a *left-block* or a *right-block*. If the position is in a *right-block* we follow the procedure as in the non-paired version above. In the second case, with the position in a *left-block*, the associated prefix sum is an overestimation of the number of bits. This requires us to subtract the bits from the location of the requested position until the end of the *left-block*. For this, instead of removing bits from a bit group on the right, we remove bits on the left, perform a popcount and subtract the results from the associated value of the *paired-block*.

The definition of L0 changes such that only *right-block* values are available. Hence L0 has only half the entries as the non-paired version.

5 $$\begin{aligned} L0[i] = \sum _{j=1}^{i\cdot 2-1} pc(b[j]) \end{aligned}$$

The *rank* operation also changes. It requires to distinguish the cases of requesting a position inside a left-, or right-block.

6 $$\begin{aligned} i_{b}&= \Big \lfloor \frac{i}{w} \Big \rfloor \end{aligned}$$

7 $$\begin{aligned} i_{l0}&= \Big \lfloor \frac{i}{2w} \Big \rfloor \end{aligned}$$

8 $$\begin{aligned} bits&= {\left\{ \begin{array}{ll} b[i_b]_{:i-i_{l0} \cdot 2w} &  \text {if } b[i_b] \text { is rb}\\ b[i_b]_{i-i_{l0} \cdot 2w+1:} &  \text {otherwise} \end{array}\right. }\end{aligned}$$

9 $$\begin{aligned} rank(i)&= {\left\{ \begin{array}{ll} L0[i_{l0}] + pc(bits) &  \text {if } b[i_b] \text { is rb}\\ L0[i_{l0}] - pc(bits) &  \text {otherwise} \end{array}\right. } \end{aligned}$$

**Fig. 2:**
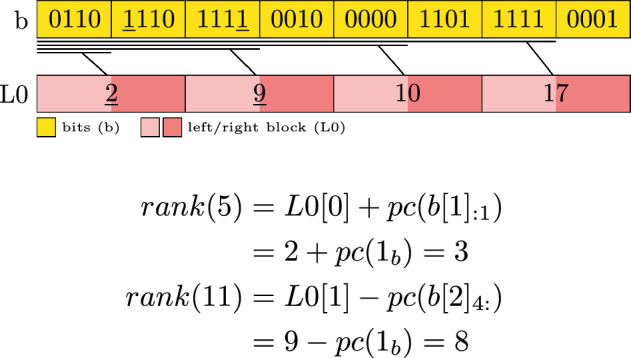
Example of a single layer paired-block bit vector with block size $w=4$. The lines between the layers indicate which section of the bits the prefix sums correspond to. Underlined values are accessed in the exemplary rank computation

Figure 2 visualizes the layout and the rank operation. *rank*(5) accesses a *right-block*, which is similar to before, while *rank*(11) accesses a *left-block* and shows how to subtract values from the next prefix sum value.

#### Two-layer

The Single-Layer approach can be extended to two (or more) layers and generalized to achieve halving the number of values stored in the auxiliary structures. However, it requires some thought. The core challenge is to change the usage of the bits and the values in *L*1 in such a way, that they can be subtracted from *L*0 to compensate for overestimation of *L*0 in half of the cases.

For each layer, to support paired-blocks the layer below must be capable of counting the bits either from the left or from the right. This depends if it’s a *left*- or *right*-block or superblock. In the single-layer implementation, this is achieved by removing the bits on the left or on the right before applying popcount.

In the implementations so far, the block values are the prefix sums from the beginning of the superblocks. In a paired-superblock version, this changes for *left-superblock* values to *postfix* sums. If the blocks are part of a *left-superblock* the value represents the number of 1 bits until the *right-superblock* starts. This is used to subtract the overcounted bits by a superblock. Blocks in the *right-superblock* stay unchanged and count the number of 1 bits from the start of the *right-superblock*.

We will first walk you through how a non-paired version is defined and implemented and then demonstrate the changes for the *paired-block* version.

The two-layer non-paired version follows the single layer version closely. It requires two adjusted helper arrays *L*0 and *L*1. Where *L*0 is a value reflecting the prefix sum value every $w_0$ bits and *L*1 are the prefix sum values every $w_1$ bits but starting to count from the last multiple of $w_0$. In all cases it must be true that $w_0$ is a multiple of $w_1$.

10 $$\begin{aligned} L0[i]&= \sum _{j=1}^{i \cdot \frac{w_0}{w_1}-1} pc(b[j])\end{aligned}$$

11 $$\begin{aligned} L1[i]&= \sum _{j=\left\lfloor \frac{i\cdot w_1}{w_0}\right\rfloor \cdot \frac{w_0}{w_1}}^{i-1} pc(b[j]) \end{aligned}$$

To access *rank* a simple access to *L*0, *L*1 and *b* has to occur:

12 $$\begin{aligned} i_{l0}&= \left\lfloor \frac{i}{w_0} \right\rfloor \end{aligned}$$

13 $$\begin{aligned} i_{l1}&= \left\lfloor \frac{i}{w_1} \right\rfloor \end{aligned}$$

14 $$\begin{aligned} bits&= b[i_{l1}]_{:i-i_{l1} \cdot w_1}\end{aligned}$$

15 $$\begin{aligned} rank(i)&= L0[i_{l0}] + L1[i_{l1}] + pc(bits) \end{aligned}$$

**Fig. 3:**
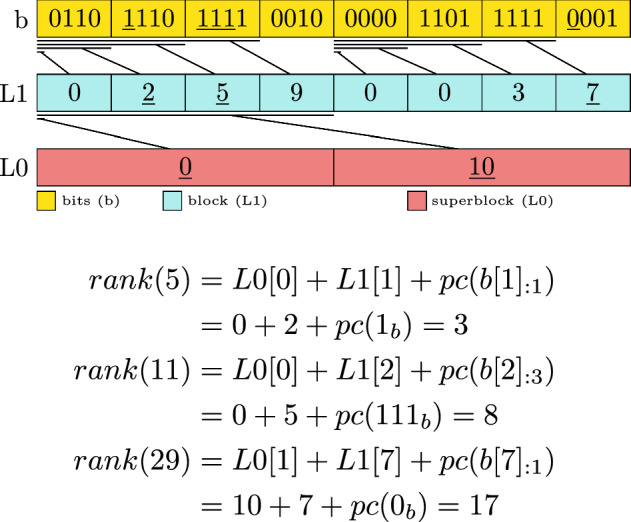
Example of a two-layer bit vector with $w_0=16$ and $w_1=4$. The lines between the layers indicate which section of the bits the prefix sums correspond to. Underlined values are accessed in the exemplary rank computation

Figure 3 shows the layout and exemplary access to *rank*(5), *rank*(11), and *rank*(29).

Next, we show the adjusted *L*0 and *L*1 computation to support a *paired-block* version. The main difference of *L*0 to before is, that it spans two $w_0$ groups. *L*1 also spans two $w_1$ groups, but its value depends on its location. When it is part of a right-superblock it counts the number of 1 bits from the start of the superblock. If it is part of a left-superblock it reflects the number of 1 bits until the right-superblock starts.

16 $$\begin{aligned} L0[i]&= \sum _{j=1}^{2i \cdot \frac{w_0}{w_1} -1} pc(b[j])\end{aligned}$$

17 $$\begin{aligned} L1[i]&= {\left\{ \begin{array}{ll} \displaystyle \sum _{j=\left\lfloor \frac{i\cdot w_1}{w_0}\right\rfloor \cdot \frac{w_0}{w_1}}^{i-1} pc(b[j]) & \text {if } b[j] \text { is rb}\\ \displaystyle \sum _{j=i}^{\left\lfloor \frac{i \cdot w_1}{w_0}\right\rfloor \cdot \frac{w_0}{w_1} + 1} pc(b[j]) & \text {otherwise} \end{array}\right. } \end{aligned}$$

To support *rank* operation with paired-blocks, we need to distinguish into multiple cases, depending on the combination of left- and right- superblock and block positions. On the bit level, it changes if we count the bits on the left or right. On the prefix sum level it modifies the values which are being added or subtracted.

18 $$\begin{aligned} i_b&= \left\lfloor \frac{i}{w_1} \right\rfloor \end{aligned}$$

19 $$\begin{aligned} i_{l1}&= \left\lfloor \frac{i}{2w_1} \right\rfloor \end{aligned}$$

20 $$\begin{aligned} i_{l0}&= \left\lfloor \frac{i}{2w_0} \right\rfloor \end{aligned}$$

21 $$\begin{aligned} pos&= i-i_{l1} \cdot 2w_1\end{aligned}$$

22 $$\begin{aligned} c&= {\left\{ \begin{array}{ll} pc(b[i_b]_{:pos}) &  \text {if } b[i_b] \text { is rb}\\ -pc(b[i_b]_{pos+1:}) &  \text {otherwise} \end{array}\right. }\end{aligned}$$

23 $$\begin{aligned} v&= {\left\{ \begin{array}{ll} L1[i_{l0}] &  \text {if } L1[i_{l1}] \text { is rb}\\ -L1[i_{l0}] &  \text {otherwise} \end{array}\right. }\end{aligned}$$

24 $$\begin{aligned} rank(i)&= c + v \end{aligned}$$

**Fig. 4:**
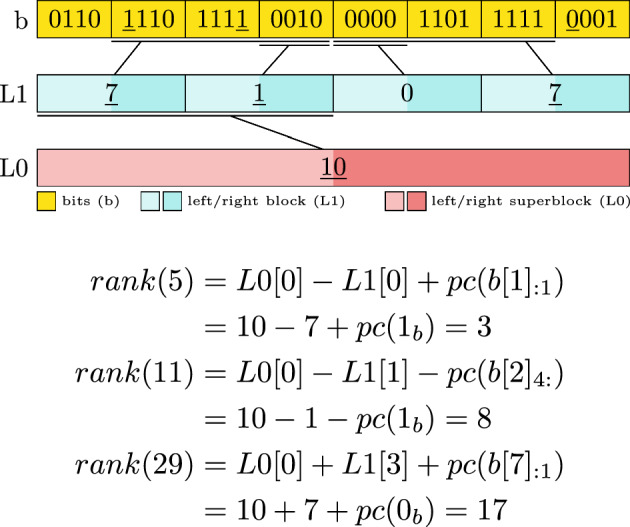
Example of a two-layer paired-block bit vector with $w_0=16$ and $w_1=4$. The lines between the layers indicate which section of the bits the prefix/postfix sums correspond to. Underlined values are accessed in the exemplary rank computation

Figure 4 shows the layout of a two-layer paired-block bit vector. When comparing Figs. 3 and 4 the halving of the support values is visible, while the bits are staying exactly the same. Additionally, we provided an example access to *rank*(5), *rank*(11), and *rank*(29). Here, the different cases when to add, subtract, count left bits or right bits are demonstrated. This approach can be extended to more layers, if desired, but will increase the number of cases that need to be distinguished.

### Mask and count

The improvement suggested in this section is specific to the hardware architecture of most modern computers.

A fast implementation of the rank operation on bit vectors requires on the lowest layer a *popcount* operation. The relevant bits of a block are stored in a variable *b* with a width of 64 bits or 512 bits, which covers a complete cache line. Before performing a *popcount* for counting the set bits to a certain position, the unwanted bits are removed. Let *j* be the number of bits we are interested in. The operation $pc(b_{:j})$ would return the number of 1 bits. The $b_{:j}$ access is usually implemented by shifting the bits to the right. We refer to this approach as *shift-and-count*. For our *paired-block* version there are multiple drawbacks of *shift-and-count*. In contrast to other implementations, our implementation of *rank* requires a case distinction to choose between left and right shifts. This distinction requires an additional branch instruction. Another disadvantage for blocks with a width of 512 bit is that we did not manage to use AVX512 efficiently, because shifting does not exist for the AVX512 instruction set. This prevented our compiler from integrating 512-bit wide *popcount* instruction, using multiple *popcount* instructions for 64-bit registers.

As an alternative, we suggest a *mask-and-count* approach which solves the above issues. The approach implements $b_{:j}$ by applying a bit mask $mask(\cdot )$ to *b*. Additionally, the mask can take into account if it is a left or right block and mask only bits on the left or the right. The masks are stored in an array and can be indexed by the requested bit position relative to the beginning of a *paired-block*, which follows that there are $2\cdot w+1$ masks where *w* is the size of a block. The values $0 \le i \le w$ reflect a shift by *i* positions to the right, as required in *left-blocks*. The values from $w \le i \le 2\cdot w$ represent a shift by $2 \cdot w - i$ to the left as required for positions in the *right-blocks*.

For picking the mask, the bit position has to be expressed as the position over a pair of blocks. A position $i \le w$ is targeted at for bits inside a left block, requiring masking bits on the left. While positions $i \ge w$ are targeted for bits located inside a right block.

**Fig. 5:**
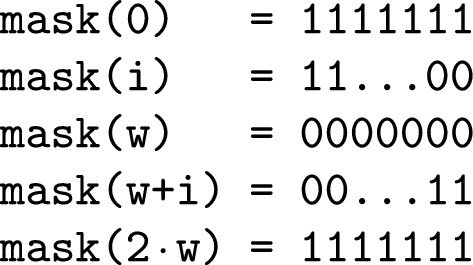
An example of the mask array for a block size of *w*. On the right side of the equation are shown binary values. A 0 indicates we are not interested and a 1 indicates we are interested in a specific position of a machine word

**Fig. 6:**
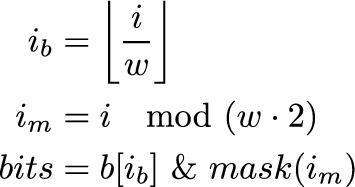
The masking part of *mask-and-count* as required for the *paired-block* implementation with 1 or 2 layers

The Fig. 5 shows the array of masks. This removes the case distinction when accessing the bits but requires an additional mask index. The adjusted access can be seen in Fig. 6.

### Rank operations on strings

Strings can also support a *rank* operation. When performing rank on strings two arguments are required. A position *i*, as for bit vectors, and a specific character *c*. The *rank* operation will count the number of characters *c* that have appeared in the first *i* positions.

The *rank* support for strings can be implemented by using any of the previously described bit vector implementations. One can create a bit vector for each character of the alphabet, which marks the position where that character appears. A *rank* request to the string is forwarded to a rank operation on the bit vector for a specific character. In the following this is called the *multiple bit vector* (mBV) implementation.

The EPR-dictionary implementation by Pockrandt et al. [1] improves on this, by removing the bits of the bit vectors and replacing them with a single compact representation of the string. This reduces the space requirements of the bits from $O(n \cdot |\Sigma |)$ to $O(n \cdot \log |\Sigma |)$. On the downside, the space required for the auxiliary data (blocks and superblocks) increases from $o(n \cdot |\Sigma |)$ to $o(n \cdot |\Sigma |\cdot \log |\Sigma |)$. The increase of the auxiliary structures stems from requiring more prefix sum values because the bits on the lowest level only span $w/\log (|\Sigma |)$ characters. To extract the bit representation from this compact string a chain of masking and bit-operations have to be performed. For details see the paper of Pockrandt et al. [1]. Parallel to our research Anderson & Wheeler [27] suggested a memory layout similar to the layout which is going to be introduced in this paper. They focused primarily on run time improvements and embedded their data structure in an FM-Index implementation with the use case of strings representing DNA or amino acid alphabets.

In this section, we want to suggest an improved variant of Anderson & Wheeler’s layout which we call *flattened bit vectors*. The initial improvement was inspired by the EPR-dictionaries and meant to reduce the run time, but mostly improved the memory usage.

**Fig. 7:**
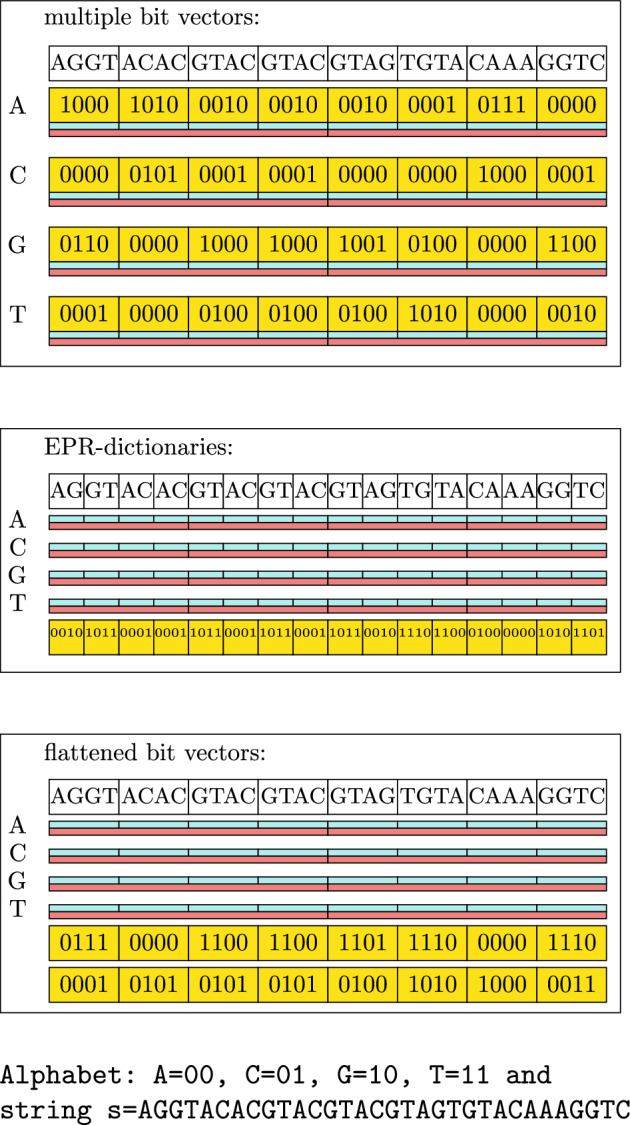
Visualization of different strings with rank support implementation and their data structures. All three require support data structures for each character (blue/red boxes). Notice that EPR-dictionaries require twice as many as the other two implementations. EPR-dictionaries and flattened bit vectors only require half the bits compared to the multiple bit vectors implementation

The core idea is to take the multiple bit vectors implementation, remove the bit layers of all bit vectors, and use a representation of the string as a replacement. Instead of using a compact representation for the string, as the EPR-dictionaries implementation does, we scatter the bits of each character over multiple words $b_i$. The number of words $b_i$ is dependent on $\lceil \log |\Sigma | \rceil$. An example of this is shown in Fig. 7. This is similar to the strided-layout by Anderson & Wheeler but our arrangement allows multi-layer auxiliary data.

From the scattered string, we can reconstruct the bits of what the bit vector would have stored. To reconstruct which position holds some character *c* it is required to apply the bitwise AND-operation over $b_i$ or the bitwise inverse of $b_i$. The final result will carry a 1 bit where *c* appeared otherwise a 0 bit.

**Fig. 8:**
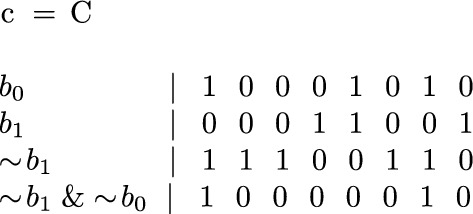
Example for an Alphabet $\Sigma =\{A, C, G, T\}$ which stores the string CAAGTACG scattered across $b_0$ and $b_1$. The expression $\sim b1~ \& ~b_0$ reconstructs all positions where ’C’ appears

If a $b_i$ is required to be inverted, depends on the character *c*. If *c* is encoded at position *i* with a 0, $b_i$ must be inverted. In our example, shown in Fig. 8 we have to invert $b_0$ and $b_1$ before AND-ing them to obtain the positions of an A. For a C we have to invert only $b_1$. In its generalized form, for any alphabet $\Sigma$ the reconstruction algorithm is shown in Listing 1.
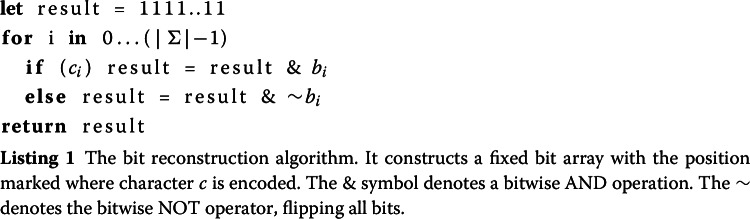

The result of the applied algorithm in Listing 1 represents the same bits as the removed bit layer of the multiple bit vectors implementation. From here on, the same procedure as with a multiple bit vectors implementation can be followed. Since the length of $b_i$ is independent of $\Sigma$ we can utilize full machine words reducing overhead for blocks and superblocks, which is a big advantage over EPR-dictionaries. The number bit operations is dependent on the size of $\Sigma$ bringing the time complexity to $O(\log |\Sigma |)$, but in contrast to Wavelet Trees, the access can be executed in parallel, making it in practice very fast on modern hardware.

## Results and discussion

As a benchmark setup, we are using an EPYC 9454 (zen4) CPU, compiling all software with clang 20.0.0 and activating the flags ’’.

### Evaluating bit vectors

Firstly, we evaluate our bit vector implementation with paired blocks and two layers, looking at the reduced space consumption and the effect on the run time. The same benchmark is also utilized to compare implementation *masking-and-counting* (which is the default) with *shifting-and-counting* (marked by the suffix ’-shift’). We also compare to the state-of-the art implementations *Rank9* by Vigna [19], the variants *SDSL-V* and *SDSL-V5* by Gog et al. [28], and lastly the implementations *FlatRank* and *WideRank* by Kurpicz [21]. To evaluate run time, we ran rank queries at random positions. For this, we used the microbenchmark library nanobench [29] which calls repeatedly rank until it reaches an accurate measurement to estimate an average call length. For memory usage we used the Linux program ’’ and checked it against the theoretically expected memory usage.

A bit vector denoted as 64/64*k* means a block size spanning 64 bits and a superblocks spanning 65,536 bits. In all our experiments we use either the combination 64/64*k* or 512/64*k* for block and superblock sizes. The block size is inspired by the available hardware support for 64 bit respectively 512 bit using AVX512. The superblock size of 64*k* was experimentally determined to give the best speed/memory tradeoff.

Table 1 shows the results of these benchmarks. The second column of Table 1 shows the space overhead in bits in percent for each implementation. For example, a value of $25\%$ indicates that the support data structure for a bit vector of length *n* adds an additional $0.25\cdot n$ bits. The remaining columns give the average time for a rank query for different text sizes.

**Table 1:** Each row shows a bit vector implementation

Implementation	Overhead	64 MB	256 MB	1GB	4GB	16GB
	in %	ns/rank	ns/rank	ns/rank	ns/rank	ns/rank
FlatRank	3.1	7.89	12.61	32.33	34.08	33.97
WideRank	3.2	5.94	10.37	25.43	26.98	26.52
SDSL-V	25.0	4.87	14.60	23.43	25.06	24.92
SDSL-V5	6.3	12.93	16.75	36.24	39.57	38.87
Rank9	25.0	5.10	14.79	24.00	25.29	25.15
64/64k	25.1	**3.89**	13.04	**20.44**	**20.72**	**20.58**
512/64k	3.2	**4.16**	**7.36**	**19.23**	**20.30**	**19.89**
Paired 64/64k-shift	12.6	6.90	10.70	**20.16**	23.24	22.89
Paired 512/64k-shift	**1.6**	13.63	17.70	28.53	30.86	30.55
Paired 64/64k	12.6	4.61	10.52	23.09	25.12	25.18
Paired 512/64k	**1.6**	5.01	**7.88**	22.65	23.89	23.59

The notation *b*/*s* denotes the size of blocks and superblocks. The second column shows the space overhead in percent. Columns 3–7 show the run time of the *rank* operation for different bit vector sizes. Best values (up to a difference of 10%) are highlighted in each column. The horizontal line separates third parties and our implementation

**Paired-Blocks**: We can see that *Paired 512/64k* requires only $1.6\%$ overhead compared to its non-paired counterpart and to *FlatRank* and *WideRank* which require $3.1\%$–$3.2\%$ overhead. While the paired version is slightly slower than the unpaired version, it is faster than *FlatRank* and *WideRank*.

Comparing the paired implementations with the non-paired implementations (not the ’-shift’ version) over different bit vector sizes shows a small speed disadvantage for paired versions. The paired version is on average about $11\%$ slower than the unpaired. In the 512-bit version, it is on average $15\%$ slower.

Our *Paired 512/64k* implementation outperforms WideRank and FlatRank in every case while requiring only half the memory overhead.

The advantage of reducing overhead from $3.1\%$ to $1.6\%$ while increasing the algorithm’s scope is limited for direct application. Looking at extended usages that compress the bit layer, or as discussed later integrated into other structures like flattened bit vectors, these small improvements can have a larger impact.

**Mask-And-Count**: The results in Table 1 also show the speed differences between the *shifted* and the *masked* version.

Comparing the speed of Paired 64/64k-shift to Paired 64/64k shows a small difference. For larger bit vectors it is in favor of the shifting version having a speed advantage of about $10\%$. This is despite the *mask* version being branch-free, while the *shift* version requires to decide if a shift to the left or right. The only exception is for the 64 MB test case, in which masking was faster.

However, the advantage of the *mask* implementation is very clear for the 512-bit case. For 512 bits, masking resolve multiple issues. As in the 64-bit case, it avoids branching to distinguish a left shift from a right shift. Additionally, it avoids the weakness of AVX512 not having a shift instruction for 512-bit wide words. This forces the compiler to emulate this using multiple 64-bit shift, which also prevented the compiler from using the available AVX512 popcount. This introduces many extra instructions and branches. The masked version solves these issues, creating a branch-free machine code with fewer instructions utilizing popcount for 512-bit words. For 512 bits we measured improvements of at least $25\%$ up to $125\%$ for the 256 MB test case. Hence, in general, we would recommend using the *mask* implementation with 512-bit block size.

### Evaluating strings

In the second part of our benchmarks and results, we want to evaluate the space reduction and run-time impact of our flattened bit vectors (fBV) and flattened bit vectors using paired-blocks (pfBV) implementation. We generated a text with 1 billion random characters divided into multiple test cases with $\sigma \in \{4, 5, 16, 21, 255\}$ different characters. Note: we choose 255 characters and not 256, because the SDSL implementation of the EPR-dictionaries cannot handle 256 or more different characters. For each case, we report the memory in bits per character and time to perform a single rank operation. As comparison, we use the Wavelet Trees and EPR-dictionaries implementation of the SDSL Library [28]. We adjusted the AwFmIndex [27] for access to its internal strings with its rank support structure. (Only available for $\sigma \in \{5, 21\}$). Additionally, we provide a custom multiple bit vector(mBV) based implementation as a reference point, of what is fastest possible, if memory is not considered a factor.

**Table 2:** Shows memory requirement and run time for different rank implementation on strings

	$\sigma =4$		$\sigma =5$		$\sigma =16$		$\sigma =21$		$\sigma =255$	
	bits/char	ns/rank	bits/char	ns/rank	bits/char	ns/rank	bits/char	ns/rank	bits/char	ns/rank
mBV	5.004	27.16	6.255	24.57	20.016	26.02	26.271	25.65	318.999	29.22
AwFmIndex	–	–	5.000	49.19	–	–	11.000	55.57	–	–
wavelet(SDSL)	2.500	93.21	**3.000**	129.68	5.000	258.58	**5.595**	297.08	** 9.995**	650.33
EPR(SDSL)	4.063	36.62	7.238	48.29	24.625	38.72	42.000	46.36	706.500	66.87
fBV 64/64k	3.004	29.83	4.255	36.63	8.016	39.01	10.271	41.97	72.000	43.04
fBV 512/64k	**2.129**	31.26	**3.161**	40.54	**4.516**	41.62	**5.677**	45.44	16.218	66.91
pfBV 64/64k	2.502	34.95	3.628	40.90	6.008	47.21	7.636	47.66	40.003	46.62
pfBV 512/64k	**2.065**	35.51	**3.081**	43.50	**4.258**	42.45	**5.339**	44.74	12.110	68.77

Five different alphabet sizes $\sigma \in \{4, 5, 16, 21, 255\}$ indicating how many distinct characters a string has are depicted. The smallest structures and values up to 10% larger are highlighted. The horizontal line separates third parties and our implementation

Table 2 lists the results of our benchmarks. Each case for $\sigma \in \{4, 5, 16, 21, 255\}$ has its own measurement of required bits and the average time it takes to perform a random rank operation.

**Fig. 9:**
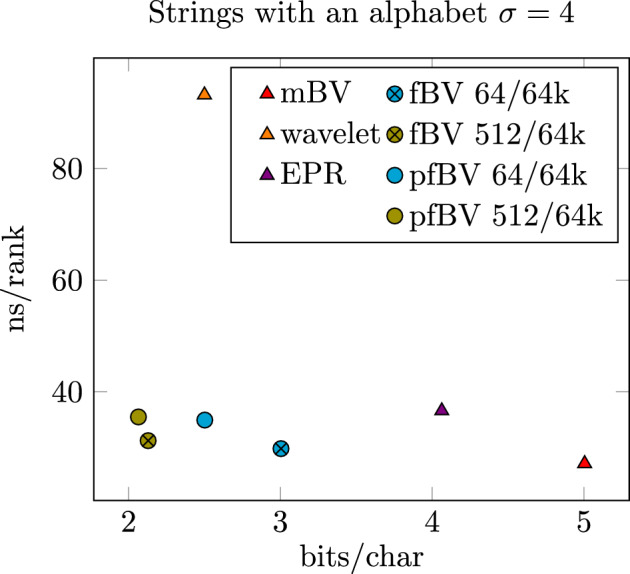
Scatter plot of strings implementation plotting memory usage against the run time of the rank operation. The lower and further left a marking, the better

Let’s first look at the cases $\sigma =4,16,255$. In the first case for $\sigma =4$ our flattened bit vectors bring a 25% (4 bits to 3 bits) memory improvement over EPR-dictionaries. Utilizing the paired block implementation with 64 bits, we have a 38% improvement(4 bits to 2.5 bits), which is on par with the Wavelet Tree implementation. Increasing the block width to 512 bits, we can even undercut Wavelet Trees memory usage by 20% only requiring 2.065–2.129 bits per character. Since the encoding of each character requires 2 bits and 4 support structures are required, the memory saving for the paired-block with 512-bit implementation are negligibly small. Only the 64-bit implementation has a large enough overhead that saving memory by using the paired-block variant has noticeable advantages. Figure 9 shows a scatter plot plotting memory against run time. It shows that our implementation of (paired) flattened bit vectors are located at the bottom left.

In the second case of $\sigma =16$, the overhead of auxiliary structures causes EPR-dictionaries to be not competitive (memory nor performance-wise) even against the default mBV implementation, while our newly suggested flattened bit vectors are competitive with Wavelet Trees. The flattened bit vectors implementation with 512-bit block size are even 10–15% smaller than Wavelet Trees while outperforming them over $6\times$ in speed.

**Fig. 10:**
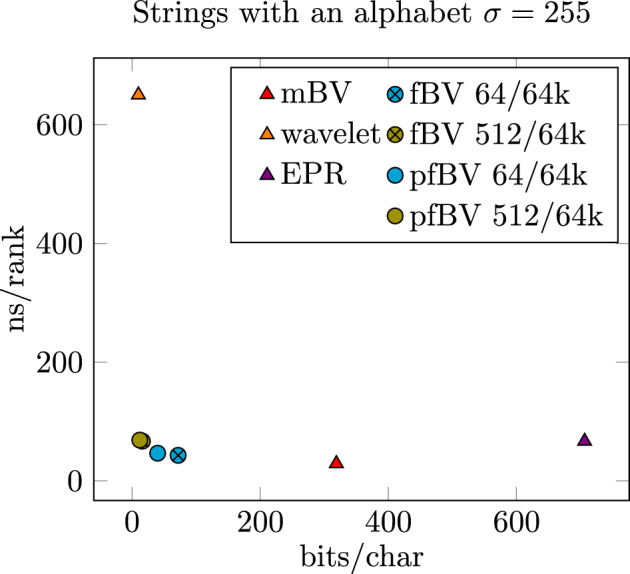
Scatter plot of strings implementation plotting memory usage against the run time of the rank operation. The lower and further left a marking, the better

For the case of $\sigma =255$, Wavelet Trees can show their advantage in memory requirements, requiring only 10bits per character. Flattened bit vectors also show their superiority over EPR-dictionaries, requiring less than 90% of the memory. The difference between paired and non-paired flattened bit vectors is particularly pronounced for such large alphabets. The paired 512-bit block version requires 25% less memory than its non-paired counterpart. In the case of 64-bit blocks, it uses even 45% less memory. For such a large alphabet, we also see the largest performance time/space tradeoff between 64-bit and 512-bit wide blocks. 512-bit blocks show an about 48% slow down while having a space overhead that is about 75% smaller. Of particular interest is the paired flattened bit vectors 512-bit version compared to the Wavelet Trees. Here the paired flattened bit vectors version requires 25% more memory but is more than $9\times$ faster. Making it a great option for performance-critical applications. This is also underlined by Fig. 10 which shows a scatter plot of memory plotted against run time. The (paired) flattened bit vectors are clustered together on the bottom left.

We also included the cases of $\sigma =5$ and $\sigma =21$, which were hard-coded in the *AWFmIndex* implementation by [27]. The columns show that the implementation requires considerably more space and is slower than all our proposed versions.

### Conclusion

We have shown a novel layout of *paired-blocks* for bit vectors with rank support. This layout reduces overhead space by $50\%$ enabling bit vectors with as little as $\approx 1.6\%$ overhead, beating the previous lowest $\approx 3.1\%$ reported by Zhou et al. [20]. Furthermore, by employing *mask-and-count* instead of the traditional *shift-and-count* approach, the *paired-blocks* reaches only slightly higher run-time compared to the non-paired-blocked versions while saving space. Our third contribution introduces a new layout *flattened bit vectors* designed for strings with rank support. This layout, combined with *paired-blocks*, requires 20% less memory than the best implementation to date, for small alphabets, while also doubling the speed. For larger alphabets, flattened bit vectors remain competitive in terms of memory to Wavelet Trees. The 512-bit paired version has only a 25% increase in space usage, while out-performing them by a factor of 9 in terms of speed.

Note that our study focused on *rank* and not *select* operation since this is the operation for strings in the FM-Index our research group is interested in. However, we do not see any inherent reason that our presented ideas should not work for efficient *select* operations.

**Recommendation:** Picking a good string with rank support implementation is a tradeoff between space usage and run time. When the application requires a binary alphabet we can recommend our 512/64k bit vectors or anything with a similar setup. While our *paired-blocks* bit vectors have a smaller space overhead it is very unlikely that an application benefits from another 1.6% space saving. For strings with non-binary alphabets, our recommendation is somewhat more differentiated. If run time is the only concern, we recommend using a multiple bit vector implementation. These can easily be derived from the previously analyzed bit vectors and outperform any other implementation. When low memory usage is important and a smaller alphabet is being used we recommend using the flattened bit vectors without paired blocks. For larger alphabets (e.g. $\sigma =255$), choosing Wavelet Trees is only recommended if memory constraints are very tight and execution time is not a consideration at all. In all other cases, we recommend using our flattened bit vectors with paired blocks. The additional saving of $25\%$ over non-paired version while losing only a little of run time execution can make a big impact. The speed improvements of up to $9\times$ clearly favor flattened bit vectors over Wavelet Trees.

## Data Availability

No datasets were generated or analysed during the current study.

## Declarations

### Source Code

Our library and benchmark is open-source and available at https://github.com/seqan/pfBitvectors/tree/v1.0.
